# Multiscale Integration of Acceleration and Jerk Sensing in the Vestibular System

**DOI:** 10.3390/audiolres16010021

**Published:** 2026-01-30

**Authors:** Leonardo Manzari

**Affiliations:** MSA ENT Academy Center, 03043 Cassino, Italy; lmanzari1962@gmail.com; Tel.: +39-3382864625

**Keywords:** vestibular system, jerk, Type I hair cells, Type II hair cells, otolith organs

## Abstract

**Background:** The vestibular system encodes head motion through specialized Type I and Type II hair cells, which differentially respond to acceleration and its temporal derivative, jerk. Molecular gradients of retinoic acid establish zonal distributions of these hair cells, prefiguring their functional specialization. **Objectives & Methods:** Here I integrate developmental, synaptic, biomechanical, and neural evidence to propose that Type I hair cells, via multimodal synaptic transmission, are particularly well suited for ultrafast detection of transient inertial deformation (jerk), whereas Type II cells play a greater role in encoding sustained acceleration through viscous-flow mechanisms. Molecular gradients of retinoic acid help establish central–peripheral zonal patterning in the otolith and canal epithelia, which in turn underlies differential mechanical and synaptic specialization rather than a simple redistribution of hair-cell types. Computational and experimental studies reveal that the vestibular organs operate in dual mechanical regimes, enabling the dynamic encoding of motion onset and continuity. In systems terms, these viscous and inertial activation modes correspond to distinct temporal filters, whose different time constants naturally give rise to distinct frequency responses. What has traditionally been described as ‘low- vs. high-frequency’ tuning therefore emerges as the frequency-domain signature of acceleration- versus jerk-sensitive pathways. **Conclusions**: This hierarchical organization elucidates the selective activation observed in clinical vestibular tests and informs novel diagnostic and rehabilitative strategies targeting specific receptor pathways. Together, these findings redefine vestibular transduction as a multimodal dynamic sensor, enhancing our understanding of balance and spatial orientation under complex motion conditions.

## 1. Introduction

The vestibular system detects head motion and spatial orientation through specialized mechanosensory epithelia—the semicircular canals and otolith organs—which transduce angular and linear accelerations into neural signals that guide reflexes and perception. Traditionally, acceleration has been considered the primary stimulus variable governing vestibular transduction. However, recent research has highlighted the importance of ‘jerk’—the rate of change of acceleration—as a key factor in detecting the onset of motion, sudden changes in velocity, and dynamic transitions. Rather than being a secondary aspect of acceleration coding, sensitivity to jerk is now understood as a fundamental property of vestibular function. Notably, jerk sensitivity appears to be an intrinsic property of vestibular organization, arising from distinct activation modes of the two major receptor classes: Type I and Type II hair cells.

Although no single study explicitly defines jerk as a primary encoded variable, convergent findings from epithelial patterning [1], multimodal Type I–calyx synaptic transmission [2,3], micromechanics of the otoliths [4], FSI simulations of the cupula [5], and clinical transient responses [6] collectively demonstrate that jerk sensitivity emerges as an intrinsic, multi-scale property of the vestibular system rather than a secondary epiphenomenon of acceleration encoding.

In this review, I use the term “jerk” to indicate the time derivative of acceleration. For rotational motion, angular jerk corresponds to the time derivative of angular acceleration (dα/dt) and primarily stimulates the semicircular canals. For translational motion, linear jerk corresponds to the time derivative of linear acceleration (da/dt) and primarily stimulates the otolith organs. Distinguishing angular from linear jerk is useful because different clinical tests preferentially engage one or the other component.

Thus, rather than simply acting as two separate ‘low- and high-frequency’ channels, these systems represent two mechanical activation modes within a unified framework. Each mode has its own characteristic time constants and therefore its own frequency response: the viscous, acceleration-dominated regime behaves as a low-pass temporal filter, while the inertial, jerk-dominated regime behaves as a fast, high-pass differentiator. Type II hair cells, more prevalent in peripheral/extrastriolar regions and innervated mainly by regular bouton afferents, contribute predominantly to the encoding of sustained, low-dynamic acceleration via conventional synaptic transmission. In contrast, Type I hair cells in central/striolar zones, enveloped by calyx-bearing irregular afferents, are particularly well suited for rapid, transient stimuli and for responding to mechanical jerk through multiple synaptic mechanisms.

This hierarchical organization is established by molecular gradients of retinoic acid [1], is reflected in the dual mechanical input regimes [4,5,6] and is integrated by neural circuitry that balances transient and sustained responses [5].

Together, these findings suggest a vestibular framework and this review synthesizes recent advances to present a unified model in which viscous and inertial activation modes coexist, enabling the vestibular system to encode both acceleration and jerk for robust equilibrium, gaze stability, and perceptual continuity under dynamically changing motion conditions.

### Clinician’s Perspective: Why a Clinician Writes This Review

As a clinician, the motivation for this review arises from a practical need: to link the anatomy and physiology of vestibular hair cells and afferent pathways with the diagnostic responses observed daily in the clinic. Contemporary and old vestibular testing—vHIT, VEMPs, SVIN, VNG, calorics, and rotatory chair—probes different dynamic regimes of the labyrinth, yet the underlying mechanistic rationale for their selective activation has remained fragmented across molecular biology, biomechanics, electrophysiology, and systems neuroscience.

Understanding why vHIT detects certain lesions while calorics detect others requires a unified framework that connects hair cell type, synaptic architecture, mechanical activation mode, and central processing. The goal of this review is therefore to create a conceptual continuum between the anatomy–physiology of vestibular encoding and the practical realities of clinical diagnosis, identifying how each test interrogates specific receptor classes, mechanical regimes, and neural pathways. In this sense, the curiosity driving this work is not academic but clinical: we aim to determine whether current diagnostic tools truly capture the full spectrum of vestibular dysfunction and to explore whether new mechanistic insights can inform more precise therapeutic and rehabilitative strategies.

## 2. Conceptual Premise and Scope of This Narrative Review

**Figure 1:**
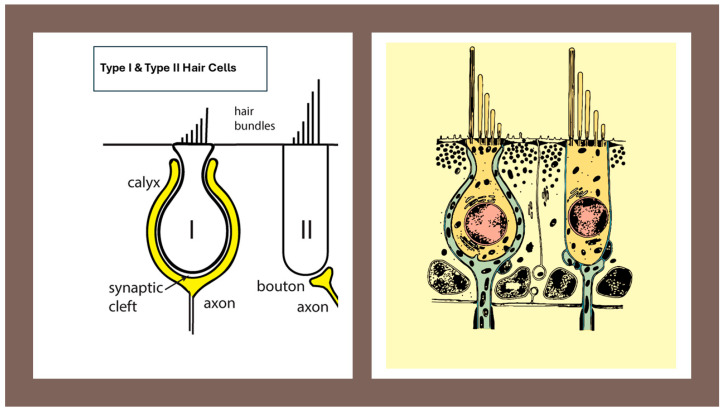
Morphological organization of the Type I vestibular hair cell and its calyx afferent. A cross-sectional representation showing the flask-shaped Type I hair cell enveloped by the calyx afferent terminal. The narrow synaptic cleft, the afferent cup, and the stereociliary bundle with kinocilium are shown. This figure provides the structural basis for understanding the multimodal transmission mechanisms described in subsequent sections. Image provided by the author.

Understanding vestibular encoding requires a multi-level perspective that spans molecular development, cellular physiology, synaptic biophysics, organ biomechanics, afferent population dynamics, and clinical neurophysiology. The present article is structured as a narrative, hypothesis-driven review, designed to integrate critical findings across these domains and reconstruct a coherent mechanistic explanation for the functional divergence between Type I and Type II vestibular hair cells (Figure 1).

This review does not aim to provide a comprehensive or systematic survey of all literature. Instead, it focuses on a curated selection of studies chosen for their mechanistic significance and explanatory power.

These include: (i) developmental patterning of vestibular epithelia [1,7], (ii) multimodal synaptic transmission at the Type I–calyx junction [2,3], (iii) vestibular biomechanics from macromechanical dual-mode operation to fluid–structure interaction [4,5,8], (iv) classical and contemporary work on afferent diversity [7,9,10,11,12,13,14], and (v) central processing mechanisms, including cross-striolar inhibition and velocity-storage dynamics [10,15,16].

The review is organized in two major components.

First, the selected studies are summarized in a way that clarifies their fundamental contributions and contextual relevance (see Box 1).

Second, these findings are synthesized to develop an integrative model that distinguishes transient, jerk-sensitive pathways from sustained, acceleration-sensitive pathways, providing a unifying explanation for the functional dichotomy between Type I and Type II hair cells and their afferent populations.

Box 1 **Glossary of key terms.**

- Jerk—Time derivative of acceleration; a measure of how quickly acceleration changes over time.
- Angular jerk—Time derivative of angular acceleration (dα/dt), mainly relevant for semicircular canal stimulation during rapid head rotations.
- Linear jerk—Time derivative of linear acceleration (da/dt), mainly relevant for otolith stimulation during translational movements or bone-conducted vibration.
- Type I hair cell—Flask-shaped vestibular hair cell, located predominantly in central/striolar zones, forming a tight synaptic complex with a surrounding calyx ending; associated with high-gain, phasic signaling (see Figure 1).
- Type II hair cell—Cylindrical vestibular hair cell, more common in peripheral/extrastriolar zones, contacted by multiple bouton endings; associated with tonic, sustained signaling (see Figure 1).
- Regular afferent—Primary vestibular neuron with nearly constant inter-spike intervals at rest and well-modulated, low-frequency responses; typically associated with Type II hair cells.
- Irregular afferent—Primary vestibular neuron with variable inter-spike intervals and enhanced sensitivity to fast, high-frequency stimuli; typically associated with Type I hair cells and calyx endings.
- Ephaptic transmission—Communication between neighboring excitable elements mediated by local electric fields in restricted extracellular spaces, rather than by classical chemical synapses.
- Non-quantal transmission—Synaptic signaling not strictly dependent on discrete vesicle release, but mediated by changes in baseline neurotransmitter concentration and/or ephaptic mechanisms.
- Viscoelastic regime—Dynamic mode in which tissue and fluid behave with both viscous and elastic components, favoring responses to sustained accelerations and low-frequency motion.
- Inertial regime—Dynamic mode in which tissue and fluid move approximately as a rigid body, so that local inertial forces dominate and responses are driven mainly by jerk and rapid changes in motion.
- Velocity storage—Central integrative mechanism that prolongs and reshapes vestibular responses to sustained rotation or low-frequency canal stimulation, contributing to low-frequency VOR and motion perception.

### 2.1. Developmental Zonation and Retinoic Acid Signaling

Retinoic acid (RA) signaling has emerged as a key developmental mechanism for establishing the regional architecture of the mammalian otolith organs [1]. In the embryonic utricle, a spatially patterned balance between RA-synthesizing and RA-degrading enzymes generates a central domain that will become the striola, surrounded by an extrastriolar region. Within this central domain, hair cells, supporting cells, the otoconial membrane and afferent innervation acquire distinctive molecular and morphological features, including a high density of complex calyceal endings and characteristic expression of Ca^2+^-binding proteins. These observations suggest that RA does not simply regulate hair-cell fate, but rather orchestrates the formation of a specialized microdomain with unique mechanical and synaptic properties.

Conditional deletion of the RA-degrading enzyme Cyp26b1 in the developing utricle disrupts this zonal organization. In the Cyp26b1 mutant, the morphological and molecular distinctions between striolar and extrastriolar regions are largely lost, and the epithelium assumes a more homogeneous, extrastriolar-like appearance. Importantly, vestibular short-latency evoked potentials (VsEPs) to transient linear acceleration are markedly reduced in amplitude and delayed in these animals, indicating that RA-dependent patterning of the central utricular zone is necessary for normal population responses to jerk-like otolithic stimuli.

It is important to note that these experiments do not directly quantify the relative proportions of Type I versus Type II hair cells in each zone and therefore do not prove a simple redistribution of hair-cell types. Rather, they provide causal evidence that RA-dependent striolar patterning generates an otolithic microdomain whose combined cellular, synaptic and mechanical specializations are required for robust, transient, high-frequency responses. In the present framework, this RA-sculpted zone is interpreted as a key substrate for jerk-sensitive otolith signaling, without implying a one-to-one mapping between hair-cell type and afferent dynamics.

### 2.2. Developmental Foundations of Transient vs. Sustained Active Vestibular Pathways

The comprehensive developmental framework described by Eatock [7,17] provides essential context for understanding how the vestibular system acquires its dual-mode encoding strategy. Her synthesis demonstrates that the ability of vestibular hair cells and their afferent pathways to encode either rapid transients (jerk) or sustained accelerations is not merely a functional divergence but the outcome of a deeply orchestrated and evolutionarily conserved developmental program. The distinction between Type I and Type II hair cells—central to this review—emerges progressively through specialized patterns of ion channel acquisition, synaptic maturation, and epithelial zonation. These developmental trajectories give rise to the fundamental dichotomy between phasic/jerk-sensitive and tonic/acceleration-sensitive vestibular encoding strategies.

### 2.3. Early Divergence of Type I and Type II Hair Cells

Vestibular sensory epithelia differentiate into diverse receptor populations early in development. Type II hair cells appear first, showing features consistent with steady-state, glutamate-driven quantal transmission. In contrast, Type I hair cells emerge later and undergo a more protracted maturation process characterized by unique morphological and electrophysiological transformations. The hallmark flask-shaped morphology of Type I cells and their envelopment by the calyx afferent terminal develop only after initial differentiation, indicating that the Type I–calyx synapse is not a default mode of transmission, but a specialized architecture built onto a more ancestral Type II–bouton template.

**Figure 2:**
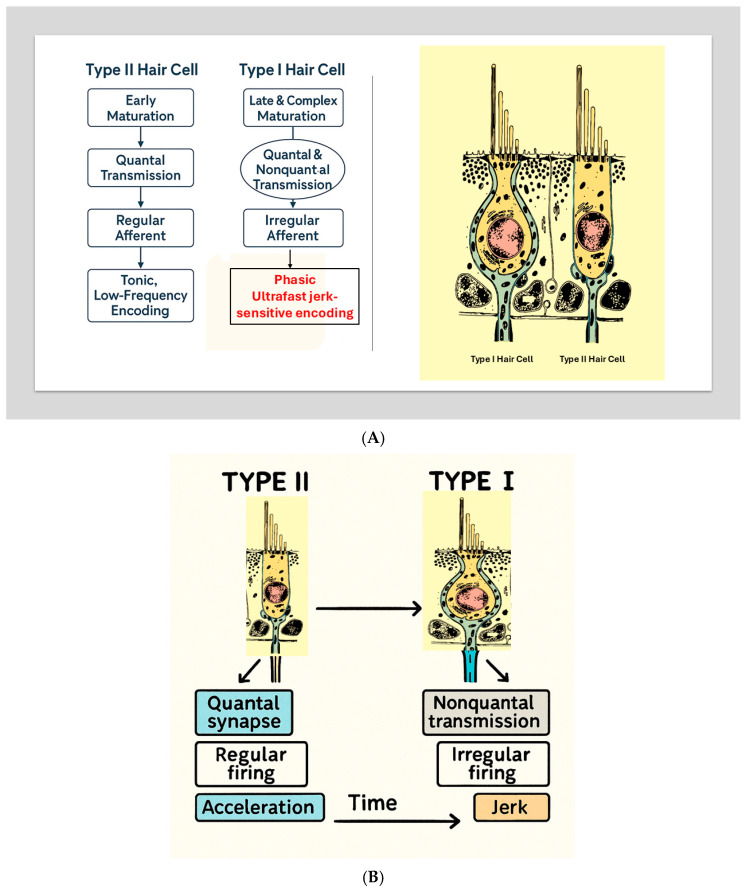
(**A**) Developmental Divergence of Type I and Type II Hair Cells. A scientific schematic illustrates the parallel but distinct developmental pathways of Type II (left) and Type I (right) vestibular hair cells. Type II cells mature early, form quantal bouton synapses, give rise to regular afferents, and support tonic encoding of low-frequency acceleration. Type I cells undergo late and complex maturation, acquire both quantal and nonquantal transmission mechanisms, develop calyx-bearing irregular afferents, and support ultrafast jerk-sensitive encoding. Adapted conceptually from Eatock (2025) [7]. (**B**) Developmental Divergence of Type I and Type II Hair Cells. Developmental schematic illustrating the divergence of vestibular hair cells into Type II (left) and Type I (right) phenotypes. Type II cells mature earlier, develop predominantly quantal synapses, regular afferent firing, and support tonic encoding of low-frequency acceleration. Type I cells undergo later and more complex maturation, acquire nonquantal transmission mechanisms (K^+^ accumulation and resistive coupling), irregular afferent firing, and support ultrafast, jerk-sensitive encoding. This developmental framework, adapted from Eatock (2025) [7], provides the biological foundation for the dual-mode vestibular model described in this review.

Eatock emphasizes that this divergence reflects adaptive specialization rather than simple variation: “Vestibular epithelia are organized into central and peripheral zones that generate afferent populations with fundamentally different encoding properties.” [7,17] (See Figure 2).

### 2.4. Zonal Patterning and Functional Territories

The division of vestibular epithelia into central (striolar) and peripheral (extrastriolar) zones constitutes one of the most robust developmental features. Type I hair cells and calyx afferents tend to mature earlier and more prominently in central/striolar regions, while Type II cells are more prevalent in peripheral extrastriolar regions, although both hair-cell types are present in all zones and their overall proportions appear broadly similar in mammalian epithelia. These zones differ in:

- Hair cell density.
- Afferent type distribution.
- Ion-channel expression profiles.
- Membrane properties.
- Mechanical coupling to otolithic or cupular structures.

The central zone becomes a high-gain, high-speed sensory domain, while the peripheral zone functions as a stabilized, low-frequency domain. This aligns perfectly with the dual-mode mechanical behavior of vestibular structures described later in this review (accelerometer vs. seismometer regimes) [7].

### 2.5. Ion Channel Maturation and the Rise of High-Speed Excitability

A defining contribution of Eatock’s chapter is the detailed description of how the electrophysiological identity of hair cells and afferents is sculpted through differential ion channel expression.

- **Type I Hair Cells**
  Type I cells progressively acquire:
  - G*_K,L_* (low-voltage activated K^+^ conductance).
  - Large basolateral K^+^ currents.
  - Fast activation/deactivation kinetics.
  - Ultra-low input resistance.
  - Short membrane time constants.
  These features enable rapid, jerk-sensitive voltage responses.
- **Afferents to Type I Hair Cells**
  Calyx-bearing afferents acquire:
  - K*_LV_* channels.
  - HCN channels.
  - NaV1.6 channels → supporting transient, persistent, and resurgent sodium currents.
  - Irregular, high-coefficient-of-variation firing patterns.

These properties reduce temporal filtering, allowing precise encoding of abrupt motion transients, consistent with phase-locking > 1 kHz documented by Curthoys et al. [8].

The RC analogy provides a simple systems-level interpretation of the KV1.8 data: Type I hair cells and their calyx afferents behave as low-τ, high-bandwidth RC filters optimized for jerk-sensitive, inertial activation, whereas Type II cells behave as high-τ, low-bandwidth filters optimized for sustained, acceleration-dominated motion. In this sense, “frequency tuning” emerges as the frequency-domain signature of their distinct temporal filters [7,15,16,18]. A quantitative RC formulation of this analogy, grounded in KV1.8 (Kcna10) data, is provided in Appendix A.

### 2.6. Emergence of Nonquantal Signaling as a Late, Specialized Adaptation

Eatock highlights a developmental milestone of exceptional importance: nonquantal transmission emerges only after the calyx terminal fully engulfs the Type I hair cell, reducing the synaptic cleft volume.

Initially, both Type I and Type II cells rely primarily on quantal glutamate release. As maturation proceeds:

- The calyx forms a tight, capacitive interface.
- Potassium gradients accumulate rapidly in the narrowing cleft.
- Direct resistive coupling becomes possible.

This gives rise to:

- Slow nonquantal transmission (K^+^ accumulation).
- Fast nonquantal transmission (resistive coupling).

Eatock writes: “Nonquantal specializations support transmission speed in two ways—eliminating the quantal delay and reducing charging time of pre- and post-synaptic membranes.” [7]. Thus, the Type I–calyx synapse is specifically engineered during development for submillisecond encoding, essential for jerk sensitivity.

### 2.7. Developmental Origin of Regular vs. Irregular Afferent Dynamics

Another key point is that afferent regularity/irregularity is not simply a response property, but an emergent developmental phenotype shaped by:

- Ion channel repertoires.
- Synapse type (calyx vs. bouton).
- Epithelial zonation.
- Membrane conductance patterns.

**Regular afferents** develop stable interspike intervals and encode slow or steady-state signals.

**Irregular afferents** mature into phasic units optimized for detecting rapid motion transients.

Eatock’s developmental framework therefore explains why jerk-sensitive and acceleration-sensitive pathways exist.

### 2.8. Development as the Blueprint for Dual-Mode Vestibular Encoding

Integrating Eatock’s developmental insights reveals a coherent hierarchy [7]:

**Sustained ensemble: bouton-rich regular afferents (peripheral/extrastriolar bias)**

- Early differentiation.
- Quantal synapses.
- Regular firing.
- Strong association with peripheral zones.
- Viscous mechanical regime.

→ ideal for steady-state acceleration.

**Transient ensemble: calyx-rich irregular afferents (central/striolar bias)**

- Late differentiation.
- Nonquantal transmission.
- Calyx synapse.
- Irregular firing.
- Strong association with central/striolar zones.
- Inertial mechanical regime.

→ ideal for rapid transients and jerk.

This developmental architecture fully supports the dual-mode vestibular model proposed in this review.

### 2.9. Macromechanical Basis: Dual-Mode Operation of the Otolith Organs

**Figure 3:**
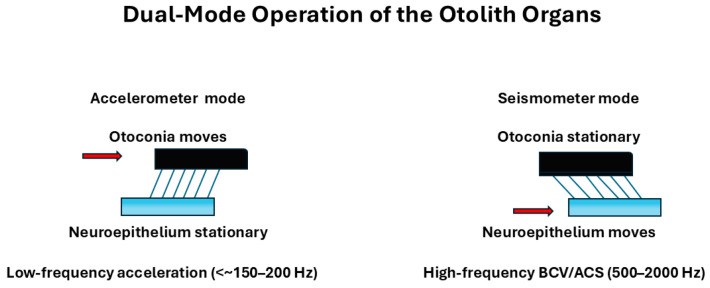
Dual-mode operation of the otolith organs. Schematic illustration of the two mechanical regimes of the otolithic organs, based on the model of Grant and Curthoys (2017) [4] and Curthoys et al. (2018, Open Access/CC BY) [23]. At stimulus frequencies below the un-damped natural frequency (~350–400 Hz), the system behaves as an accelerometer (**left**): head linear acceleration causes the neuroepithelial base to accelerate while the otoconial mass lags behind, so that the relative shear between otoconia and neuroepithelium (and the embedded hair bundles) is proportional to linear acceleration. At stimulus frequencies well above the undamped natural frequency (**right**), the otolithic system operates as a seismometer: the otoconial mass remains nearly stationary due to its inertia, whereas the neuroepithelium vibrates, and the resulting relative shear is proportional to neuroepithelial displacement. This high-frequency “seismometer mode” provides the mechanical substrate for activation of otolithic afferents by bone-conducted vibration and air-conducted sound during VEMP stimulation. Red arrows indicate the moving element in each regime.

Grant and Curthoys [4] described the otolith organs as systems capable of operating in two distinct mechanical modes, depending on the characteristics of the stimulus. At low frequencies, the otolith organs function like accelerometers, detecting and encoding static head tilt and sustained acceleration (Figure 3).

At higher frequencies, the interaction between the otoconial layer and the sensory epithelium causes the system to behave more like a seismometer, which is sensitive to rapid changes in motion and thus preferentially encodes jerk—the rate of change in acceleration. This shift in mechanical behavior forms the basis for the physiological distinction between the two main hair cell types:

- Sustained ensemble: bouton-rich, more regularly discharging afferents (peripheral/extrastriolar bias) that preferentially encode steady or slowly varying head motion (velocity and acceleration envelopes) and contribute prominently to tonic vestibulo-ocular and vestibulo-spinal reflex components.
- Transient ensemble: calyx-rich and dimorphic afferents with more irregular discharge (central/striolar and canal-core bias) that emphasize rapid changes in motion (high-frequency components, onset/offset transients) and therefore exhibit jerk-rich sensitivity when vibration drives rapid alternations of acceleration. Type I hair-cell biophysics and the Type I–calyx complex provide key high-bandwidth specializations for this ensemble; however, both Type I and Type II hair cells are present across zones, and pathway-level specialization is best described in terms of zonal location and afferent terminal architecture rather than an anatomically segregated Type I-only channel.
- From the earliest days of vestibular research, investigators have sought to resolve—at the smallest possible (“ultra-small”) scale—how the peripheral apparatus converts motion into neural signals. Pioneering in vivo observations of cupular motion in the living labyrinth (Steinhausen, 1927) and the first quantitative physical formulations of semicircular canal fluid–cupula mechanics (van Egmond, Groen & Jongkees, 1949) established the classical flow-driven framework that still underpins modern viscoelastic descriptions of canal transduction [19,20]. More recently, this macromechanical foundation has been complemented by evidence that the vestibular periphery can generate highly synchronized, ultrafast neural output, consistent with transmission mechanisms operating on sub-millisecond timescales [21]. Clinically, this duality maps onto a dissociation between tests dominated by transient/jerk-rich stimuli versus sustained low-frequency canal dynamics—exemplified in Ménière’s disease, where caloric responses can be markedly reduced despite near-normal vHIT gains [22].

### 2.10. Synaptic Microphysiology and Ionic Coupling

**Figure 4:**
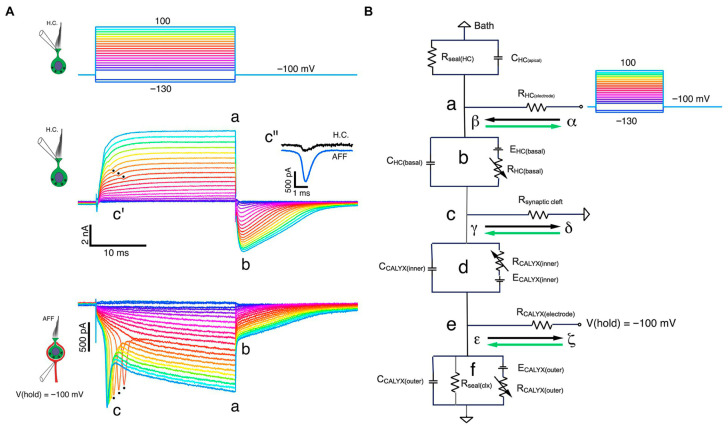
Multimodal transmission at the Type I–calyx synapse (adapted from Contini et al., 2024) [3]. Simultaneous dual recordings from a Type I vestibular hair cell and its calyceal afferent demonstrate the presence of three modes of transmission: (1) quantal glutamatergic release, (2) slow non-quantal K^+^ accumulation in the synaptic cleft, and (3) fast bidirectional resistive coupling mediated by cleft resistance. Panel (**A**) shows the voltage-clamp responses of hair cell and calyx under depolarizing steps, including retrograde action currents. Panel (**B**) illustrates the equivalent circuit model that explains fast orthograde and retrograde interactions. Reprinted with permission from Contini, Holstein & Art (2024), Frontiers in Neurology [3].

Electrophysiological and modeling studies (Contini, Holstein, and Art) [3] show that transmission at the Type I hair cell–calyx synapse operates through multiple modes. In addition to conventional quantal glutamatergic release at ribbon synapses, the cleft can support slower non-quantal depolarization mediated by K^+^ accumulation, and a very fast non-quantal resistive coupling pathway in which bidirectional currents flow through the ohmic resistance of the narrow synaptic cleft. This resistive mechanism is distinct from classical ephaptic field effects; in the revised manuscript we therefore use the term “resistive coupling” throughout when referring to this ultra-fast pathway (Figure 4).

### 2.11. Empirical Validation Using Laser Doppler Vibrometry

Recent work by Pastras and colleagues [8] has provided a valuable quantitative link between otolith macromechanics and ensemble neural responses. Using laser Doppler vibrometry ((type 8338—Brüel & Kjær, Denmark), these authors measured the motion of the utricular macula while simultaneously recording vestibular compound action potentials (vCAPs) during bone-conducted vibration and other transient stimuli. A key finding is that vCAP amplitude scales most consistently with macular velocity across a wide range of stimulation paradigms, rather than with displacement or acceleration per se. When responses are plotted against the kinematics of the head or skull, apparent shifts between “acceleration-like” and “jerk-like” sensitivity emerge because macular velocity relates differently to bone motion in different temporal regimes of a multi-degree-of-freedom mechanical system.

To interpret these data, Pastras et al. [8] used a two-degree-of-freedom model showing that the utricle behaves as a simple inertial sensor that responds to acceleration transients, with emergent dual regimes when responses are expressed in terms of the imposed skull kinematics. For short, high-frequency stimuli, macular velocity is closely related to the jerk of bone motion, whereas for longer, lower-frequency stimuli it follows bone acceleration more directly. In the present review, I therefore use “acceleration- versus jerk-sensitivity” as a shorthand for these effective stimulus regimes at the macular and afferent level, rather than as evidence for a binary switch at the level of the hair-cell transduction apparatus. The Pastras data [11] are most naturally interpreted as demonstrating dual inertial regimes of the otolith macromechanics, which can then be differentially exploited by downstream synaptic and neural circuitry.

### 2.12. Neural Circuit Integration and Reflex Dynamics

Curthoys and colleagues have expanded the understanding of how peripheral jerk sensitivity translates into central vestibular processing. Cross-striolar and commissural inhibitory circuits further enhance transient responses, providing a central gain mechanism for jerk encoding [6,7,9,10]. These circuits ensure that the central nervous system can rapidly initiate compensatory reflexes such as the vestibulo-ocular and vestibulo-spinal reflexes upon abrupt motion onset.

## 3. Integrative Framework: Transient vs. Sustained Vestibular Pathways

The evidence reviewed across developmental, synaptic, mechanical, and systems-level studies converges on a dual-mode organizational principle that recasts vestibular encoding as two complementary pathways optimized for different dynamic regimes of motion.

(1) Transient ensemble: jerk-sensitive, calyx-rich irregular afferents (central/striolar bias)

The first pathway is characterized by its specialization for encoding rapid motion transients and jerk, the time derivative of acceleration. This mode is driven by:

- The characteristic organization of central/striolar zones, with dense Type I–calyx and dimorphic innervation.
- The emergence of nonquantal and resistive synaptic transmission enabling sub-millisecond responsiveness.
- Inertial cupular deformation demonstrated by fluid–structure models, independent of sustained endolymph flow.
- Otolith seismometer-mode mechanics at high frequencies.
- The irregular firing patterns supported by low-impedance membranes and specialized sodium and potassium channels.
- Central circuits that bypass velocity storage and amplify onset responses.

Together, these features define a high-speed vestibular detection system capable of encoding abrupt changes in motion with extreme temporal precision.

(2) Sustained ensemble: acceleration-sensitive, bouton-rich regular afferents (extrastriolar bias)

The second pathway supports the encoding of steady or slowly varying acceleration, including gravity and prolonged head movements. This channel is supported by:

- Early-maturing Type II hair cells prevalent in peripheral extrastriolar zones;
- Classical quantal synaptic transmission with higher input resistance;
- Viscous endolymph flow dynamics in the semicircular canals at low frequencies;
- The accelerometer mode of otolith mechanics;
- Regular afferent discharge patterns well-suited to representing sustained stimuli;
- Integration within the velocity-storage network, extending the temporal representation of motion.

This pathway forms the basis for tonic vestibulo-ocular and postural control, as well as the Importantly, the distinction between these two pathways does not reflect separate “frequency bands,” but rather distinct activation mechanisms. The transient system is activated by inertial, flow-independent mechanical inputs that preferentially stimulate Type I regions. The sustained system is driven by viscous, flow-dependent mechanics activating Type II regions.

This multiscale integrative view bridges hair cell biology, biomechanics, afferent physiology, and clinical testing, offering a conceptual framework that unifies decades of disparate findings into a single coherent model of vestibular encoding. stable perception of orientation.

### 3.1. Anatomical and Clinical Integration of Jerk Sensitivity

**Figure 5:**
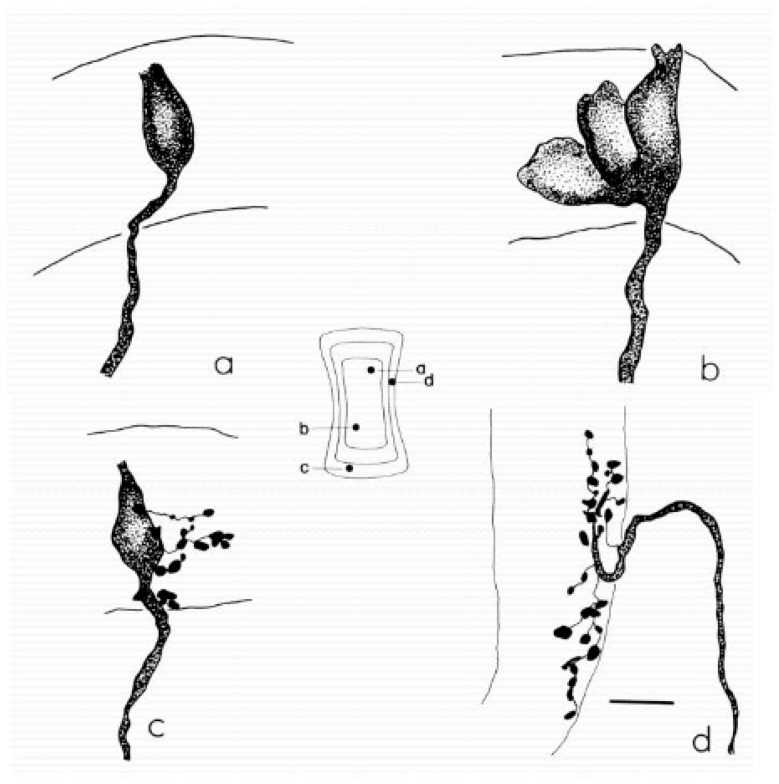
Examples of the main classes of semicircular canal afferents in the chin-chilla. (**a**) Calyx afferent with a single simple calyx contacting one type I hair cell in the central zone of the crista. (**b**) Calyx afferent with a complex calyx that envelopes several adjacent type I hair cells. (**c**) Dimorphic afferent giving rise to a calyx ending on a type I hair cell and multiple bouton endings on neighboring type II hair cells. (**d**) Bouton afferent terminating exclusively in bouton endings on type II hair cells in the peripheral zone. The central schematic shows a flattened re-construction of the horizontal canal crista, indicating the entry points (**a**–**d**) of each afferent and the approximate extent of the sensory epithelium. Scale bar, 10 µm. Reprint from Fernández, Baird & Goldberg (1988), J. Neurophysiol. 60:167–181 with permission [13].

The anatomical organization of vestibular afferents provides the structural framework for understanding jerk sensitivity across peripheral and central vestibular pathways. Classic work by Fernández, Baird and Goldberg [13] demonstrated that vestibular nerve fibers can be classified as calyx, bouton-only and dimorphic units, with characteristic distributions within the cristae and maculae (Figure 5).

Central or striolar regions are characterized by dense calyceal and dimorphic innervation and by a higher prevalence of irregularly discharging afferents with phasic, high-gain responses to rapid motion. Peripheral or extrastriolar regions contain more bouton-only fibers with regular resting discharge and more linear, low-frequency response properties. This zonal organization is conserved across canal cristae and otolith maculae and provides a natural anatomical substrate for dynamic specialization.

Importantly, quantitative ultrastructural studies in mammals indicate that Type I and Type II hair cells are present in both central and peripheral zones, with broadly similar Type I/Type II ratios across regions. What changes most strikingly between zones is the density and complexity of calyx and dimorphic endings, the distribution of bouton-only afferents, and local features of the synaptic and supporting architecture. The dynamic differences between central/striolar and peripheral/extrastriolar responses are therefore best understood as emerging from the combination of hair-cell types, afferent morphologies and zonal mechanics, rather than from a simple numerical dominance of one hair-cell class. Within this context, central/striolar, calyx-rich, irregularly discharging ensembles form a predominantly jerk-sensitive mode, optimized for rapid changes in motion, whereas more peripheral, bouton-rich, regularly discharging ensembles form a predominantly acceleration-sensitive mode, optimized for sustained, low-frequency signals. Any mapping between clinical tests and “Type I” or “Type II” pathways must thus be understood as referring to these overlapping ensembles and zones, not to pure, isolated hair-cell types. Goldberg [11] extended these findings by identifying distinct projection patterns for regular and irregular afferents within the vestibular nuclei. Irregular afferents, primarily originating from Type I hair cells, terminate on large, proximal dendrites of vestibular nucleus neurons, forming fast-conducting channels optimized for transient motion.

Regular afferents tend to receive a larger contribution from bouton terminals and exhibit more linear, sustained encoding across a broad dynamic range. Importantly, in mammals most vestibular afferents are dimorphic, combining calyceal contacts on Type I hair cells with bouton contacts on Type II hair cells. Therefore, discharge regularity and frequency-response specializations map most consistently onto zonal location and terminal architecture (bouton-only versus calyx-rich/dimorphic), rather than onto hair-cell type alone. Throughout the revised manuscript we accordingly emphasize “central/striolar, calyx-rich irregular ensembles” versus “peripheral/extrastriolar, bouton-rich regular ensembles,” while treating Type I/Type II specializations as contributory biophysical factors.

This dual anatomical organization forms the basis for the separation of fast, jerk-sensitive pathways from slower, sustained integrative circuits.

Curthoys and Zee [6] further elucidated that the velocity storage mechanism acts as an adaptive temporal filter that extends the response duration to sustained stimuli but remains bypassed by the direct, irregular afferent-driven pathway. The direct (Type I) channel is thus primarily responsible for rapid, high-frequency responses to jerk stimuli, while the indirect (Type II) channel maintains longer-lasting perceptions of motion through the vestibulo-cerebellar network.

Clinically, this anatomical and physiological dichotomy is reflected in specific diagnostic tests. Vibration-induced nystagmus (SVIN), vestibular-evoked myogenic potentials (VEMPs), and vestibular short-latency evoked potentials (VsEPs) selectively assess the irregular afferent pathway dominated by Type I hair cells and calyx endings. These tests are highly sensitive to transient linear or angular accelerations and are thus key measures of jerk pathway integrity. Conversely, caloric testing, rotational chair studies, and head-shaking nystagmus (HSN) primarily probe the regular afferent pathway and the velocity storage system, reflecting low-frequency and sustained-motion responses.

In clinical practice, selective dysfunctions of these pathways can be distinguished by their physiological signatures. Patients with loss of irregular afferents typically exhibit absent VEMP and VsEP responses but preserved caloric function, while those with regular afferent deficits demonstrate prolonged post-rotatory nystagmus and disrupted motion continuity perception.

Recent evidence [6,18] emphasized that skull vibration and skull vibration-induced nystagmus (SVIN) predominantly engages irregular afferents and largely bypasses velocity storage, as evidenced by the marked reduction, or frequent absence, of afternystagmus. This interpretation remains valid, but clinical recordings show that a brief afternystagmus may occasionally be observed after strong bone-conducted stimulation. Within the present framework this behavior can be understood as the result of three partially overlapping mechanisms. First, the bypass of velocity storage is graded rather than all-or-none, so a small fraction of the response may still be routed through central integrative networks, generating a short-lived stored component; this is consistent with the established role of velocity storage as the central generator of afternystagmus [16] and with its dependence on otolith–canal integration and graviceptive context [17]. Second, intense SVIN stimuli can partly recruit dimorphic and even Type II units, adding a more sustained, acceleration-biased discharge that decays over hundreds of milliseconds. Third, local viscoelastic “tail” dynamics within the labyrinth imply that abrupt cessation of a high-frequency stimulus is followed by a brief relaxation phase of tissues and fluid. The occasional afternystagmus observed after SVIN is therefore compatible with a mainly jerk-driven test that nonetheless interacts in a graded fashion with slower mechanical and central integrative processes.

In summary, classical microanatomical mapping [11,12,13,19,20], central projection analyses and systems-level work together support a multiscale distinction between fast, phasic and slower, tonic vestibular signaling. Central/striolar regions, with their high density of calyx and dimorphic endings and their prevalence of irregular discharge, contribute a powerful jerk-sensitive component, whereas more peripheral, bouton-rich regions with regular discharge support sustained, low-frequency encoding and strong coupling to velocity storage. Dimorphic afferents bridge these regimes by sampling both zones. In the present review, I interpret clinical dissociations between tests as reflecting the preferential engagement of these overlapping ensembles, while explicitly recognizing that Type I and Type II hair cells are intermingled across zones and do not form strictly segregated “jerk” and “acceleration” islands.

### 3.2. Functional Implications: Dynamic Activation Modes Rather than Frequency Segregation

The classical view of vestibular hair cell function has often been framed in terms of frequency selectivity, with regular and irregular afferents assumed to encode low- and high-frequency motion, respectively. However, recent multiscale evidence indicates that this distinction is an oversimplification. Rather than representing separate frequency bands, Type I and Type II hair cells and their associated irregular and regular afferents operate as distinct activation channels within a single mechanical and neural continuum. The vestibular labyrinth does not primarily operate as a set of isolated frequency bands but instead decomposes head motion according to the temporal dynamics of the stimulus specifically, whether it is sustained or transient. In systems terms, this means that distinct mechanical activation modes (viscous vs. inertial) implement different temporal filters, whose characteristic time constants then appear experimentally as different frequency sensitivities.

At the biophysical level, the semicircular canal and otolith systems behave as viscoelastic structures capable of switching between two operating regimes:

(1) A viscous-flow regime, in which endolymph displacement leads to steady deflection of the cupula or otolithic membrane.
(2) An inertial-deformation regime, in which differential inertia between the endolymph and sensory structures generates rapid elastic strain without sustained flow.

This dual-mode architecture and its relationship to commonly used clinical vestibular tests is summarized in Table 1 within the Clinical and Translational Implications section.

In systems terms, the viscous-flow regime behaves like a low-pass temporal filter that integrates head acceleration over time, whereas the inertial regime behaves more like a high-pass differentiator that emphasizes rapid changes in motion. What appears experimentally as “low- vs. high-frequency tuning” is therefore the frequency-domain manifestation of these two underlying temporal filters.

The transition between these regimes depends not on stimulus frequency but on the rate of change in acceleration, that is, on jerk. Consequently, Type II hair cells, located peripherally and coupled to regular afferents, are preferentially activated during the viscous-flow regime, encoding sustained accelerations. In contrast, Type I hair cells, concentrated in central and striolar zones, and endowed with large low-voltage–activated K^+^ conductances, implement a fast, low-impedance temporal filter that is tuned to the inertial regime and responds selectively to transient motion or abrupt changes in acceleration, with broader bandwidth and minimal phase lag [20,22].

This reinterpretation carries significant physiological implications. It unifies previously fragmented observations of vestibular responses across different tests and species by recognizing that the apparent “high-frequency” behavior of irregular afferents is in fact sensitivity to transient mechanical events, not to sinusoidal frequency per se. It also provides a conceptual framework for integrating molecular, mechanical, and electrophysiological data: the same anatomical substrate can express multiple operational states depending on the temporal dynamics of the stimulus. In effect, the vestibular system acts as a dynamic-mode transducer, whose different temporal filters naturally give rise to distinct frequency responses.

Clinically, this perspective explains why diagnostic tests that employ brief, high-jerk stimuli—such as the Head Impulse Test (vHIT), VEMPs, VsEPs, and SVIN—selectively activate Type I–dominated pathways, while caloric and low-frequency rotational tests probe Type II–mediated, viscous-flow mechanisms [22]. Recognizing this dual-mode organization resolves the apparent mismatch between test frequency and receptor tuning observed in clinical practice. It also emphasizes that vestibular disorders should be evaluated not solely by their spectral characteristics but by their disruption of mechanical activation modes within the same sensory system.

In summary, vestibular function is better described as a dynamic balance between two activation regimes—one sustained and viscous, the other transient and inertial—each of which can be rigorously modeled as a temporal filter with a characteristic frequency-domain signature. The primary distinction lies in the underlying activation mode (acceleration vs. jerk), while “low-” and “high-frequency” behavior emerge as derived properties of these filters, rather than as independent organizing principles.

### 3.3. From Frequency Bands to Temporal Filters: Distinct Activation Modes of Type I and Type II Hair Cells

The classical framework describing vestibular hair cell function has long emphasized frequency-dependent sensitivity as the primary distinguishing feature between Type I and Type II receptors. Type II hair cells and their regular afferents were considered low-frequency, tonic encoders of sustained acceleration, whereas Type I hair cells and their irregular afferents were regarded as high-frequency, phasic encoders of transient stimuli. While this dichotomy has been historically useful, it no longer captures the full complexity of vestibular transduction. The fundamental distinction between these receptor types lies not in fixed ‘frequency bands’ per se, but in their activation mode—that is, in the physical and physiological mechanism by which motion is transduced into neural signals [23]. These activation modes can be rigorously formalized as different temporal filters, each with its own characteristic frequency response. Emerging evidence from developmental biology, biomechanics, and synaptic physiology demonstrates that the fundamental distinction between these receptor types lies not in the frequency band of stimulation, but rather in their activation mode, in the physical and physiological mechanism by which motion is transduced into neural signals. The semicircular canal, therefore, does not host two separate frequency filters, but rather a single viscoelastic continuum capable of operating in two dynamic regimes. In the viscous-flow (accelerometric) regime, the system behaves as a low-pass integrator of head acceleration; in the elastic–inertial (jerk) regime, it behaves as a high-pass differentiator that emphasizes rapid changes in motion. These two regimes provide the mechanical substrate for the functional dichotomy between Type II–dominated, acceleration-sensitive pathways and Type I–dominated, jerk-sensitive pathways, which differ in their temporal filters and, consequently, in their apparent frequency tuning. For a compact quantitative link between time constants (τ), bandwidth, and KV1.8-dependent conductances, see Appendix A [24,25].

#### 3.3.1. Anatomical and Mechanical Foundations

A foundational way to compare vestibular afferent dynamics is to measure responses to sinusoidal head motion across frequency. In this framework, the response of an afferent can be described by two complementary quantities: gain (how strongly firing modulation scales with stimulus amplitude) and phase (whether firing leads or lags the stimulus). When plotted across frequency, these gain–phase curves provide a frequency-domain “fingerprint” of the underlying time constants and filters implemented by the receptor–afferent ensemble. Importantly, what is often described as “frequency tuning” is typically the *frequency-domain expression* of these temporal filters, rather than evidence for separate hard-wired “frequency channels.” [4,5,7]. Across species and end organs, a consistent pattern emerges: irregular afferents show higher gain and larger phase lead at higher frequencies, whereas regular afferents display flatter gain and smaller phase shifts over the same frequency range [5,7]. In canal afferents, for example, irregular units tend to increase sensitivity as frequency rises and to exhibit pronounced phase advance, consistent with a more phasic / differentiator-like behavior; regular units remain more tonic/integrator-like, preserving a relatively stable representation of slower components of motion [5,7,26]. Similar principles apply to otolith afferents when translation or linear acceleration is used as the driving stimulus: irregular units more strongly emphasize rapid changes, while regular units better preserve sustained components [7]. Crucially, these dynamic differences are not explained solely by hair-cell class (Type I vs Type II), because in mammals both hair-cell types are present across zones and most afferents are dimorphic, receiving convergent bouton (Type II) and calyceal (Type I) inputs [3,4]. Instead, the strongest organizing principle for dynamics is the zonal/architectural ensemble: central/striolar (or canal-core) regions are enriched in calyx-rich terminals and irregular discharge, whereas peripheral/extrastriolar regions contain a greater proportion of bouton-only terminals and regular discharge, yielding distinct aggregate filtering properties at the systems level [3,4]. In this view, “irregular vs regular” is not merely a descriptive label of spike-train variability; it is a functional signature of different synaptic geometries, membrane conductances, and coupling conditions that collectively determine bandwidth and phase behavior [3,4,5,7]. Mechanistically, several factors likely contribute to these contrasting transfer functions. First, irregular afferents are commonly associated with calyx-bearing or calyx-dominated terminals and with receptor/terminal specializations that support fast transduction and rapid spike timing [3,4]. Second, differences in ion-channel complements across receptor–afferent microcircuits can reshape effective membrane time constants, thereby altering the frequency dependence of gain and phase [3,5]. Third, the mechanical interface between end-organ structures and the surrounding labyrinthine fluids introduces frequency-dependent constraints that interact with neural filtering—so that the measured response reflects both mechanical coupling and synaptic/electrical filtering [3,7]. These sinusoidal-response signatures matter directly for the present review because high-frequency vibration is mechanically “jerk-rich” (Section 2): as stimulus frequency increases, temporal derivatives of motion (particularly changes in acceleration) are increasingly emphasized. Afferent ensembles with inherently higher bandwidth and stronger phase advance—classically, those dominated by calyx-rich irregular afferents in central/striolar territories—are therefore expected to be preferentially recruited by such jerk-rich stimulation, whereas bouton-dominated regular ensembles are expected to contribute more strongly to sustained, low-frequency regimes [1,3,4,5,7].

#### 3.3.2. Biophysical and Synaptic Distinctions

At the cellular level, the activation mode of each receptor type is defined by its synaptic architecture, ion-channel complement, and resulting membrane time constants—in other words, by the RC properties that determine how the cell filters time-varying inputs.

Type II hair cells form conventional bouton synapses with glutamatergic quantal transmission mediated primarily by AMPA-type receptors. Their basolateral membranes exhibit higher input resistance and slower channel kinetics, supporting graded, sustained neurotransmitter release that integrates over hundreds of milliseconds. Functionally, this corresponds to a slower temporal filter with longer effective time constants and narrower bandwidth, ideal for tonic encoding of continuous motion and low rates of change.

In contrast, Type I hair cells are enveloped by a single calyx afferent terminal that creates a confined synaptic cleft characterized by low resistance and strong capacitance coupling. Type I cells acquire large low-voltage–activated K^+^ conductances (gK,L) and other fast currents, which drastically lower input resistance and shorten the membrane time constant. Calyx-bearing afferents, in turn, express K*_L**V**_*, HCN, and NaV1.6 channels that support rapid, irregular firing and minimal temporal smoothing.

Studies by Contini, Holstein, and Art [2,3] revealed that this Type I–calyx arrangement supports at least three complementary transmission mechanisms:

Quantal release, providing conventional glutamatergic excitation.

Slow non-quantal coupling, mediated by K^+^ accumulation in the narrow cleft.

Fast non-quantal resistive coupling, which is enabled by the narrow, high-resistance synaptic cleft and the large facing membrane areas of the Type I hair cell and calyx. On the timescale of tens of microseconds, current can flow bidirectionally through the ohmic resistance of the cleft, providing an ultra-fast pathway for transmission of rapid receptor-potential changes. This resistive mechanism is distinct from classical ephaptic coupling, which refers to field effects mediated by changes in extracellular potential without direct current flow between partners. We now reserve “ephaptic” for this historical field-effect usage and use “resistive coupling” for the mechanism described in Contini et al.

Together, these features produce submillisecond charging times and an effectively high-pass synaptic transfer function. In systems terms, the Type I–calyx complex behaves as a biological differentiator: a fast temporal filter that converts changes in mechanical force into nearly instantaneous voltage changes. Thus, the difference between Type I and Type II hair cells is not merely a matter of “bandwidth” in an abstract sense, but of synaptic physics and time constant design: one system optimized for speed and temporal differentiation, the other for steady-state integration.

#### 3.3.3. Molecular and Developmental Determinants

The developmental blueprint of vestibular epithelia further supports this mechanistic dichotomy, but again at the level of zonal specialization rather than strictly segregated hair-cell types. As discussed earlier in this review, Ono et al. showed that retinoic acid (RA) signaling via the degrading enzyme Cyp26B1 is required to establish a morphologically and molecularly distinct striolar domain within the utricle, surrounded by an extrastriolar region. When Cyp26B1 is conditionally deleted, these zonal distinctions are largely flattened and the epithelium assumes a more homogeneous, extrastriolar-like appearance; in parallel, vestibular short-latency evoked potentials (VsEPs) to transient linear acceleration are markedly reduced in amplitude and delayed. In systems terms, RA-dependent striolar patterning appears necessary for a robust fast component of the otolithic response, but the experiments do not directly demonstrate a simple redistribution of Type I versus Type II hair cells. I therefore interpret these developmental data as showing that molecularly defined zones with distinctive mechanical and synaptic architectures are required for normal jerk-sensitive otolith signaling, without implying that only Type I cells contribute to such responses.

Developmental analyses by Eatock [7] further show that Type II pathways mature earlier, with simpler ion channel repertoires and quantal synapses, whereas Type I–calyx complexes emerge later, acquiring nonquantal transmission and low-impedance membranes optimized for rapid transients. Regular and irregular afferent firing patterns thus arise as developmental phenotypes of slow vs. fast temporal filters, rather than as arbitrary differences in noise statistics.

#### 3.3.4. Functional and Clinical Consequences

Recognizing that Type I and Type II receptors implement distinct temporal filters and activation modes, rather than strictly segregated frequency bands, helps resolve several paradoxes in vestibular physiology and clinical testing.

What is often described as “high-frequency” sensitivity of irregular afferents is more accurately interpreted as sensitivity to rapid transients and high jerk, not to continuous sinusoidal oscillations per se. 

Thus, a plausible implementation of the canal response to high-frequency vibration is the combination of (i) a slow, viscous, acceleration-sensitive filter dominated by bouton-rich, more regular afferent ensembles (peripheral/extrastriolar bias), and (ii) a fast, inertial, transient/jerk-rich filter dominated by calyx-rich/dimorphic, more irregular afferent ensembles (central/striolar and canal-core bias).

Type I hair-cell biophysics and the Type I–calyx synapse contribute importantly to the high-bandwidth behavior of the central irregular ensemble, but the pathway-level specialization is best attributed to the zonal/terminal ensemble rather than to a strictly segregated Type I-versus-Type II pathway.

VEMPs, VsEPs, and SVIN responses can be reinterpreted as assays of the inertial activation mode and its associated high-speed temporal filters, rather than as mere “high-frequency” tests.

Clinically, this distinction shifts the interpretive focus from abstract frequency ranges to which temporal filter and mechanical regime are being interrogated. Lesions that compromise Type I hair cells, calyx coupling, or the molecular patterning of central/striolar zones will preferentially impair jerk-sensitive, fast reflexes—even when tonic balance and low-frequency perception remain relatively preserved. Conversely, disorders targeting Type II populations or extrastriolar zones may spare transient responses while degrading sustained motion perception and velocity storage.

In this framework, abnormal patterns such as normal vHIT with pathological calorics, or the reverse dissociation, naturally reflect selective disruption of parallel temporal filters and activation modes, rather than inexplicable “frequency-specific” weaknesses of particular tests [22].

#### 3.3.5. Toward a Unified Conceptual Framework

Redefining Type I and Type II hair cells as components of a dual-mode, multi-scale temporal filtering system helps integrate perspectives from molecular development, synaptic microphysiology, organ-level mechanics, and systems neuroscience.

Rather than functioning as separate, hard-wired filters along a frequency axis, the two receptor classes represent complementary operational states of a single mechanical–neural continuum:

A slow, viscous, acceleration-sensitive filter dominated by bouton-rich regular afferents (peripheral/extrastriolar bias), and a fast, inertial, transient/jerk-rich filter dominated by calyx-rich irregular afferents (central/striolar bias).

A fast, inertial, jerk-sensitive filter (Type I/irregular pathway).

In this view, the classical “low- vs. high-frequency” description is retained but demoted to a derived property: it is simply how these temporal filters appear when probed with sinusoidal stimuli. The primary organizing principle becomes mode-dependent activation—viscous vs. inertial, integrator vs. differentiator—implemented by distinct time constants, conductances, and synaptic architectures.

This paradigm shift—from frequency-dependent tuning to temporal filtering and activation modes—captures the essence of vestibular computation: a dynamic system that extracts both the magnitude (acceleration) and the temporal derivative (jerk) of motion through parallel but integrated pathways. In practical terms, Type II cells provide the “steady eye” of the system, stabilizing orientation during sustained acceleration, whereas Type I cells act as “motion onset detectors,” signaling abrupt changes in head movement.

Together, these complementary filters allow the vestibular system to operate seamlessly across the entire temporal landscape of motion—from seconds-long rotations to millisecond transients—without invoking rigid frequency partitions. The same logic extends beyond the otolith organs to the semicircular canal cristae, where central, calyx- and dimorphic-rich regions and more peripheral, bouton-rich regions implement homologous viscous and inertial modes, providing a unified anatomical and functional framework for dual-mode vestibular transduction.

### 3.4. Extension of Zonation Principles to the Semicircular Canal Crista

A similar pattern of zonal specialization is evident in the semicircular canal cristae, which can be regarded as the canal analog of otolith striola and extrastriola. In the central region of each crista, afferent innervation is dominated by calyx and dimorphic fibers with irregular discharge and pronounced sensitivity to rapid head accelerations. Towards the periphery, bouton-only afferents with regular resting discharge and more linear, low-frequency response properties become more prevalent. As in the otolith organs, Type I and Type II hair cells are distributed across both central and peripheral regions; what differs most is the pattern of afferent terminations, the three-dimensional arrangement of hair bundles and supporting structures, and the local mechanical environment.

In the dual-mode interpretation adopted here, the central canal crista, with its concentration of calyceal and dimorphic afferents and its position in the canal’s mechanical strain field, is particularly well suited to operate in an inertial, jerk-dominated regime during rapid head impulses. The periphery, with its richer population of bouton-only, regularly discharging fibers and its stronger coupling to central velocity-storage mechanisms, is better suited to encode sustained angular velocity and low-frequency accelerations. This does not imply that central units are insensitive to low-frequency motion or that peripheral units do not respond to high-frequency stimuli; rather, it emphasizes that canal mechanics and afferent architecture together create a continuum of dynamic tuning, with central regions providing a high-gain phasic component and peripheral regions providing a more tonic, integrative component.

From a clinical perspective, these zonal specializations offer a natural explanation for why high-jerk tests such as the video Head Impulse Test primarily reveal deficits in the central, jerk-sensitive canal components, whereas caloric irrigation and low-frequency rotational chair testing preferentially probe the more sustained, acceleration- and velocity-dependent components that rely heavily on velocity storage. The same canal can thus appear “normal” under one class of tests and “abnormal” under another, depending on which dynamic mode and which zones of the crista are preferentially engaged.

### 3.5. Proposed Model: Inertial Cupular Deformation and Type I Hair Cell Activation in the Absence of Endolymph Flow

*Note: The following section presents a theoretical hypothesis that remains to be experimentally validated. It aims to provide a conceptual framework for understanding Type I hair cell activation in the absence of sustained endolymph flow*.

Recent evidence invites a reconsideration of the classical view that endolymphatic flow is the exclusive mechanical event responsible for activating hair cells within the semicircular canals. Traditional models [19,20] describe the cupula as a viscoelastic partition displaced by sustained endolymph flow during angular acceleration. However, such models cannot fully explain the responsiveness of Type I hair cells and their associated irregular afferents to transient, high-frequency, or vibrational stimuli conditions under which no steady endolymph current is present [4,18,19,20,21].

Recent fluid–structure interaction (FSI) models demonstrate that, during rapid angular accelerations, the cupula can undergo substantial inertial deformation even in the absence of measurable endolymph flow. This deformation is driven by the differential inertia between the endolymph and the cupular mass, generating a transient pressure gradient that produces an asymmetric, S-shaped deflection of the cupula [6]. Crucially, the region of maximal elastic strain is consistently localized at the central crista—precisely where Type I hair cells and irregular afferents are concentrated—indicating that these receptors are preferentially stimulated under high-jerk conditions. The timescale of this deformation is in the order of milliseconds, matching the fast onset dynamics and irregular discharge patterns characteristic of Type I afferents [4,12,13].

Anatomically, the concentration of Type I hair cells and calyx endings in the central (crista) zones of the ampulla supports this hypothesis. These regions are structurally optimized to experience the largest instantaneous changes in mechanical force during head jerk or bone-conducted vibration. Conversely, Type II hair cells, distributed peripherally, are more responsive to steady-state deflection associated with sustained endolymph flow and acceleration. This spatial segregation suggests a functional division of labor:

- Type I/irregular afferents operate as dynamic detectors of jerk and transient motion.
- Type II/regular afferents act as static encoders of continuous acceleration.

The inertial deformation hypothesis is consistent with the transition from acceleration-dominant to jerk-dominant regimes observed experimentally in vestibular compound action potentials [21] and with the capacity of irregular afferents to phase-lock to high-frequency vibrations (>250 Hz) in the absence of sustained endolymph flow [18].

It also provides a mechanical rationale for the existence of Type I cells within the cupula-bearing ampullary crests: rather than encoding slow, steady flow, these receptors specialize in detecting the rate of change in mechanical force acting on the cupula—effectively functioning as differentiators within the system’s mechanical hierarchy.

If validated experimentally, this model would expand the current understanding of semicircular canal transduction, positioning the cupula–endolymph complex not only as a low-frequency accelerometer but also as a high-pass inertial sensor capable of encoding jerk. Such dual functionality would parallel the “accelerometer–seismometer” duality already described for the otolith organs [4], providing a unified framework for interpreting vestibular responses to both sustained and transient head motion.

### 3.6. Unified Model of Jerk Encoding

Integrating evidence from developmental biology, cellular physiology, biomechanics, and systems neuroscience, a unified, multiscale model of jerk encoding emerges that redefines the vestibular system as a temporally hierarchical motion sensor.

Rather than functioning as a simple accelerometer, the labyrinth operates as a dynamic continuum in which molecular patterning, synaptic microarchitecture, mechanical duality, and neural circuitry converge to encode both acceleration and its temporal derivative jerk.

At the molecular level, the spatial gradients of retinoic acid (RA) regulated by Cyp26B1 [1] establish a developmental blueprint that divides vestibular epithelia into distinct central (striolar) and peripheral (extrastriolar) domains.

These molecular territories prefigure the later functional specialization of the sensory epithelia: striolar regions, with a high density of calyceal and dimorphic endings and a prevalence of irregular afferents, contribute much of the structural substrate for jerk-sensitive, transient encoding; conversely, extrastriolar regions, richer in bouton-only, regularly discharging fibers, maintain sustained, acceleration-dependent signaling.

A comparable zonation pattern is observed in the semicircular canal cristae, where the central crest, with its concentration of calyx and dimorphic terminations, corresponds to the mechanical focus of cupular deformation, while more peripheral regions, with a higher proportion of bouton-only afferents, respond primarily to prolonged viscous flow.

Thus, a single molecular and anatomical principle—the central–peripheral asymmetry—underlies both otolithic and canal transduction.

At the cellular level, the Type I–calyx synaptic complex serves as a multimodal transmission unit uniquely optimized for transient activation.

As shown by Contini, Holstein, and Art [2,3], this junction supports three concurrent communication modes:

1. Conventional quantal glutamatergic signaling;
2. Slow non-quantal transmission mediated by K^+^ accumulation in the synaptic cleft;
3. Fast non-quantal or resistive coupling enabling resistive current flow between the hair cell and its calyx.

This combination of ionic and resistive mechanisms produces a submillisecond response that allows calyx-bearing afferents to follow rapid deformation events of the cupula or macular membrane.

In effect, the Type I–calyx complex functions as a biological differentiator, converting changes in mechanical force into instantaneous neural discharge—the neural correlate of jerk detection.

At the organ level, the labyrinth operates in dual mechanical regimes [6,7,8,9,10,11,12,13,14,15,16,17,18].

In the viscous-flow regime, steady endolymph movement deflects the cupula or otolithic membrane, activating Type II hair cells and regular afferents tuned to continuous acceleration.

In the inertial-deformation regime, the differential inertia between the endolymph and the cupular mass generates brief elastic strain even without measurable flow, selectively stimulating Type I hair cells and irregular afferents.

This mechanical shift—from viscous to elastic–inertial behavior—defines the physical substrate of jerk encoding and explains why high-jerk stimuli such as head impulses preferentially engage Type I receptors.

At the neural circuit level, inhibitory and commissural interactions within the vestibular nuclei [6] enhance transient signaling from irregular afferents while shaping the sustained dynamics of regular ones.

Accordingly, vibratory stimuli are expected to preferentially recruit central/striolar, calyx-rich irregular ensembles that emphasize acceleration transients, rather than the more regular bouton-dominated ensembles that encode sustained motion.

This does not imply an exclusive hair-cell-type pathway: both Type I and Type II hair cells contribute to mammalian afferents, but the biophysical specializations of the Type I–calyx complex are especially well suited to support high-bandwidth encoding within the central irregular ensemble.

This architecture forms a dual-channel system: one phasic and high-gain, optimized for motion onset and jerk detection; and one tonic and integrative, optimized for sustained motion and orientation stabilization.

Together, these molecular, cellular, mechanical, and circuit-level mechanisms define a unified model of jerk encoding in which the vestibular system captures both the magnitude and the rate of change in head motion through complementary activation modes.

The otolith organs and semicircular canals thus operate as dual-function sensors—low-frequency accelerometers and high-pass inertial detectors—whose coordinated outputs ensure both stability and agility of reflexive and perceptual responses.

This hierarchical organization allows the vestibular system to detect abrupt, transient motion while maintaining continuous awareness of steady acceleration, an essential property for equilibrium, gaze stabilization, and spatial orientation.

#### Phase-Locking of Irregular Afferents as Evidence for Jerk Encoding

**Figure 6:**
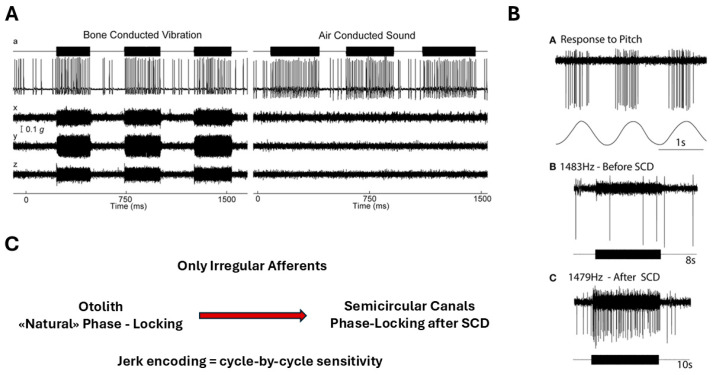
Phase locking of irregular vestibular afferents during high-frequency stimulation. (**A**) Otolith irregular afferent: time series and circular phase histogram of a utricular irregular neuron responding to 500 Hz bone-conducted vibration (BCV). The neuron does not fire on every cycle, but spikes occur within a narrow range of stimulus phase angles, demonstrating precise phase locking. Reprinted from Curthoys IS, Vulovic V. Vestibular primary afferent responds to sound and vibration in the guinea pig. Experimental Brain Research. 2011;210:347–352, with permission from Springer Nature [26]. (**B**) Anterior semicircular canal irregular afferent after superior canal dehiscence (SCD): before SCD, the afferent shows no response to high-frequency air-conducted sound (ACS). After SCD, the same neuron exhibits strong cycle-by-cycle phase-locked firing, consistent with enhanced mechanical coupling of acoustic/vibratory energy to the canal. Reprinted from Curthoys IS. The new vestibular stimuli: sound and vibration—anatomical, physiological and clinical evidence. Experimental Brain Research. 2017;235:957–972, with permission from Springer Nature [27]. (**C**) Original schematic (L. Manzari): conceptual comparison of phase-locking capability in irregular utricular versus irregular anterior semicircular canal afferents. Otolith afferents can exhibit cycle-by-cycle phase locking to high-frequency BCV/ACS under normal conditions, whereas canal afferents typically require a third-window mechanism (e.g., SCD) to show comparable phase-locked responses.

Irregular vestibular afferents demonstrate a remarkable ability to phase-lock to high-frequency bone-conducted and air-conducted stimuli, with synchronization exceeding 1 kHz [18] (Figure 6).

This degree of temporal fidelity is unparalleled in other sensory systems and indicates that these afferents are intrinsically tuned to encode ultrafast mechanical events. Such phase-locking is made possible by the multimodal signaling architecture of the Type I hair cell–calyx synapse, where quantal, non-quantal K^+^ accumulation, and resistive coupling together support submillisecond response timing [2,3]. These findings integrate naturally with biomechanical evidence showing that transient inertial deformation of the cupula can occur without sustained endolymph flow [6], providing a coherent mechanistic explanation for why irregular afferents constitute the primary jerk-sensitive channel of the vestibular system.

### 3.7. Supporting Evidence from Computational Biomechanics

**Figure 7:**
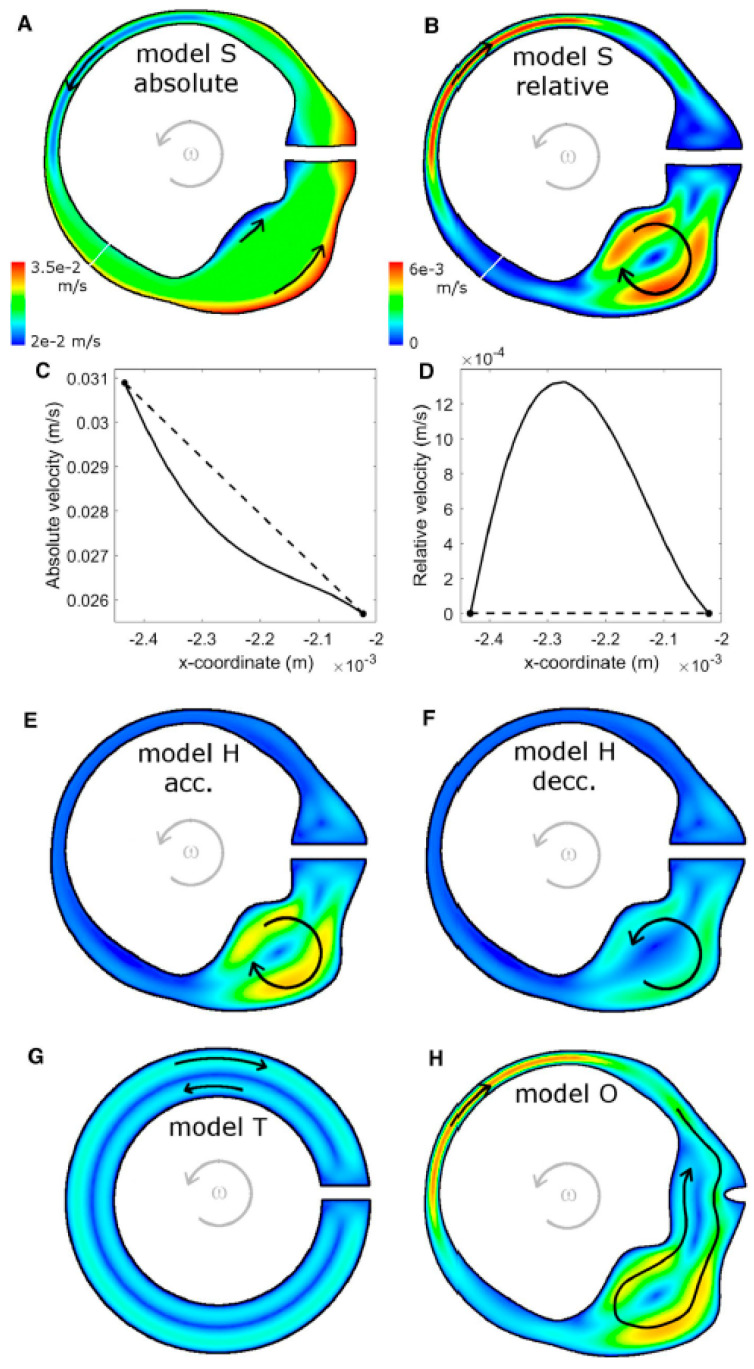
Endolymph velocity in the XY-plane of the lateral semicircular canal–utricle system during an “alarm turn” head rotation (t = 0.08 s; panel F at t = 0.16 s). (**A**,**B**) Model S: absolute (**A**) and relative (**B**) endolymph velocity fields. (**C**,**D**) Velocity magnitude along the narrow-duct cross-sections indicated by the white lines in (**A**,**B**), plotted in the absolute (**C**) and relative (**D**) reference frames. (**E**,**F**) Relative velocity field in the human geometry with rigid cupula (model H) during angular acceleration (**E**) and deceleration (**F**). (**G**) Relative endolymph velocity in the torus geometry (model T). (**H**) Relative endolymph velocity for the human geometry with open ampulla (model O). The same color scale is used for all relative-velocity maps. Gray arrows indicate the direction of head rotation; black arrows schematically indicate the direction of endolymph flow. Relative velocities are small, so individual fluid particles do not move completely from utricle to ampulla or vice versa during the maneuver. Re-produced from Goyens et al., Biomechanics and Modeling in Mechanobiology, 2019, with permission [5].

Recent computational models of the human semicircular canal–cupula system have provided strong biomechanical support for the inertial deformation hypothesis proposed in the preceding sections. Using advanced three-dimensional fluid–structure interaction (FSI) simulations, Goyens, Pourquie, Poelma, and Westerweel (2019) [5] demonstrated that during short, high-jerk angular head impulses (<250 ms), the cupula undergoes an asymmetric S-shaped deformation characterized by maximal mechanical strain localized at the central crest of the crista ampullaris (Figure 7).

In mammals, Type I and Type II hair cells are distributed across both striolar and extrastriolar zones, with only modest gradients in Type I–Type II ratios reported in several species. The more salient zonal distinction for dynamics is the density and complexity of afferent terminals: central/striolar regions are characterized by higher calyx density and a greater prevalence of irregular discharge dynamics, whereas peripheral/extrastriolar regions show relatively more bouton-only innervation and more regular firing. Because many afferents are dimorphic and integrate inputs from both hair-cell types, we interpret high-frequency, jerk-rich sensitivity primarily as a property of zonal/afferent ensembles—within which Type I/calyx specializations contribute key high-bandwidth mechanisms—rather than as an anatomically segregated Type I-only pathway.

Importantly, the FSI simulations showed that the cupular relaxation time constant (~3 s) is an order of magnitude longer than the duration of these transient impulses. Consequently, the cupula behaves as an elastic–inertial structure, not as a viscous flow detector, during the timescale of the Head Impulse Test.

Under these conditions, the localized strain peaks predicted by the model are expected to selectively stimulate Type I hair cells within the central crista while exerting minimal shear forces on Type II cells located peripherally.

These results provide quantitative mechanical validation for the proposed inertial deformation model, linking the structural dynamics of the cupula to the physiological selectivity of Type I activation.

Moreover, the findings directly support the clinical interpretation of the video Head Impulse Test (vHIT) as a selective probe of Type I hair cell and irregular afferent function.

Residual and partially recovering VOR responses after superior or posterior canal plugging provide an additional test case for this dual-mode framework. Several clinical series have reported that head impulse gain is often not reduced to zero after plugging and may even improve over months despite radiological evidence of a permanently occluded canal. Rather than contradicting the mechanics of the system, these findings are compatible with a transition from a flow-dominated, low-frequency regime to an inertial, jerk-dominated regime. In the presence of a distal or incomplete plug, high-jerk head impulses can still induce local inertial deformation of the cupula–endolymph complex and of the canal wall, without requiring substantial bulk flow along the lumen. Plugging would therefore primarily abolish sustained, low-frequency convection and strongly attenuate acceleration- and velocity-based responses, while leaving a residual phasic component that can be recruited by very rapid head impulses. Central adaptive mechanisms likely contribute to the gradual improvement of VOR gain, but the persistence of a measurable, phase-appropriate response is more easily understood if local inertial activation is allowed than under a purely flow-based model.

By integrating computational biomechanics with physiological and clinical evidence, the work of Goyens et al. [6] establishes a direct mechanical bridge between the jerk-dominant dynamics predicted by theory and the selective afferent activation observed in experimental and clinical settings.

## 4. Clinical and Translational Implications

The recognition that jerk sensitivity is a defining feature of vestibular transduction carries profound clinical and translational implications.

Different vestibular tests probe distinct mechanical and cellular regimes of the same system: the video Head Impulse Test (vHIT) and vibration- or sound-evoked responses such as VEMPs and SVIN primarily assess the transient, inertial mode mediated by Type I hair cells and irregular afferents, whereas caloric and low-frequency rotational tests evaluate the sustained, viscous mode dominated by Type II hair cells and regular afferents.

To make this distinction directly applicable to everyday practice, Table 1 summarizes the dominant stimulus dynamics, predominant receptor populations and degree of velocity-storage engagement for commonly used vestibular tests.

This conceptual framework clarifies long-standing clinical discrepancies in which patients show normal caloric responses but abnormal vHIT gains, or vice versa—reflecting selective involvement of distinct receptor classes rather than frequency-dependent limitations of the tests themselves.

Disorders that disrupt Type I cell integrity, calyx coupling, or retinoic acid–mediated patterning are expected to selectively impair jerk-sensitive reflexes even when tonic balance mechanisms remain preserved. Conversely, degenerative or metabolic conditions affecting Type II cells may leave transient responses intact while compromising sustained motion perception and velocity storage. This latest proposed dissociation between preserved transient canal responses and impaired sustained motion processing is consistent with clinical observations. McGarvie et al. (2015) [22] described patients with unilateral Ménière’s disease who showed markedly reduced caloric responses despite essentially normal horizontal canal gains on the video head impulse test and they attribute this condition to hydropic expansion of the semicircular canal duct. This condition represents a clinical example of dissociation in which the low-frequency components of canal function, and thus sustained motion perception and velocity storage, are selectively impaired, whereas high-frequency transient responses remain relatively preserved, consistent with the hypothesis of greater involvement of pathways mediated by type II cells.

These insights establish a cell-specific diagnostic logic that links physiological responses directly to underlying receptor populations and their mechanical operating modes. Beyond diagnosis, the inertial deformation model suggests new directions for vestibular prosthesis and rehabilitation design.

Electrical or mechanical stimulation protocols incorporating controlled jerk components could preferentially recruit dynamic, Type I–dominated pathways, thereby improving the temporal fidelity and perceptual realism of artificial vestibular stimulation. Similarly, tailored rehabilitation strategies that include transient head impulses or vibratory stimuli might facilitate selective plasticity within jerk-sensitive circuits, accelerating recovery after peripheral lesions.

By connecting biomechanics to clinical function, jerk sensitivity thus provides a unifying translational framework linking peripheral mechanics, neural circuitry, and therapeutic innovation.

## 5. Conclusions

Jerk sensitivity emerges as a fundamental organizing principle of vestibular function, integrating molecular patterning, synaptic microphysiology, biomechanics, and systems-level circuitry into a coherent dynamic framework.

From the developmental establishment of zonal identities [1,2,3,4,5,6,7] to the multimodal synaptic coupling of Type I hair cells [2,3], and the biomechanical dual-mode operation of the labyrinth [4,6,7,8,9,10,11,12,13,14,15,16,17,18], converging evidence supports a hierarchical design optimized to detect the temporal derivative of acceleration.

The selective activation of Type I hair cells during inertial deformation events—as demonstrated by computational and clinical findings—links the mechanical, cellular, and reflex domains of vestibular physiology into a unified model of transient motion encoding.

This integrative perspective reframes the vestibular labyrinth not as a frequency-filtered system, but as a multimodal dynamic sensor capable of operating seamlessly across tonic (acceleration) and phasic (jerk) regimes.

Future research combining computational biomechanics, in vivo imaging, and molecular genetics will be essential to test the inertial deformation hypothesis quantitatively and to explore its implications for sensory prosthetics and motion-perception theory.

Ultimately, understanding how the vestibular system encodes jerk may redefine clinical assessment, inspire new rehabilitation paradigms, and guide the next generation of bio-inspired artificial balance systems.

### Author’s Note on Review Scope (Narrative Review Statement)

This article is a narrative, integrative review that does not aim to exhaustively survey all the literature on vestibular physiology but instead synthesizes key experimental, biomechanical, developmental, and clinical findings to develop a coherent conceptual model. Because the core aim is theoretical integration rather than systematic evidence aggregation, the structure of the review reflects the author’s clinical perspective and the need to bridge basic science and diagnostic practice.

## 6. Reference Acknowledgment

The references cited in this manuscript were selected to build a coherent, multiscale framework of vestibular function, spanning molecular patterning, cellular and synaptic physiology, organ-level mechanics, and central/clinical integration. Developmental and molecular insights were drawn primarily from Ono et al. (2020) [1], whose work on retinoic acid degradation and Cyp26B1 established the zonal blueprint (striolar vs. extrastriolar, central vs. peripheral) that underlies the differential distribution of Type I and Type II hair cells and their afferents.

A central conceptual contribution to this review derives from the developmental synthesis presented by Eatock (2025) [7], whose work provides the most comprehensive and mechanistically grounded account of how mammalian vestibular hair cells diverge into the functionally distinct Type I and Type II phenotypes. Her analysis of epithelial zonation, ion-channel maturation, and synaptic differentiation offers essential context for understanding the emergence of the dual-mode vestibular encoding model proposed in this manuscript. In particular, Eatock’s demonstration that nonquantal transmission and calyx formation arise as late, specialization-driven developmental events strongly support the interpretation of Type I pathways as intrinsically optimized for phasic, jerk-sensitive signaling, while Type II pathways mature earlier into tonic, acceleration-sensitive sensors. Her chapter also clarifies how regular and irregular afferent discharge patterns emerge from molecular and electrophysiological differentiation, providing a developmental foundation for the dual-channel organization of vestibular afferents central to modern clinical testing.

For these reasons, Eatock (2025) [7] serves as a critical developmental reference that anchors the present review’s integrative framework, linking ontogeny, electrophysiology, biomechanics, and clinical vestibular diagnostics.

The cellular and synaptic substrate for transient, jerk-sensitive encoding was derived from the experimental series by Contini, Holstein, and Art (2017–2024) [2,3], which redefined the Type I–calyx junction as a multimodal synapse—combining quantal glutamatergic release, slow non-quantal K^+^ accumulation, and fast resistive coupling. These studies were integrated with classic work by Fernández, Baird, and Goldberg (1971–1988) [11,12] and the conceptual synthesis of Eatock (2025) [7] which together established the anatomical and physiological basis of Type I vs. Type II receptors, irregular vs. regular afferents, and dimorphic units spanning all sensory zones.

The mechanical and biomechanical context was informed by Grant and Curthoys (2017) [4], Pastras et al. (2023) [21], whose analyses of otolith and canal mechanics support the dual-mode “accelerometer–seismometer” operation central to this review. The fluid–structure interaction model of Goyens et al. (2019) [5] provided quantitative validation for inertial cupular deformation, demonstrating localized strain maxima at the central crista and thereby supporting the proposed mechanism of selective Type I activation in the absence of sustained endolymph flow.

At the systems and circuit level, the anatomical and functional organization of regular and irregular pathways was based on the foundational work of Fernández & Goldberg and the central projection analysis by Goldberg (2000) [14], complemented by Uchino’s description of cross-striolar and commissural inhibitory circuitry (Uchino 2004; Uchino & Kushiro 2011) [9,10]. These studies delineate how central inhibitory networks sharpen transient responses and shape vestibulocollic and vestibulo-ocular reflex pathways. The role of the velocity-storage integrator and its pharmacological and functional modulation was drawn from Cohen and colleagues (1987–2019) [15,16], who identified velocity storage as a GABA-sensitive central mechanism that prolongs low-frequency responses and underlies afternystagmus and motion sickness. Contemporary work by Curthoys and collaborators (e.g., Curthoys et al. 2019; Pastras et al. 2023; Curthoys & Zee 2025) [6,18,21] links these peripheral and central mechanisms to clinical tests such as vHIT, VEMPs, VsEPs, SVIN, calorics, and rotatory chair responses.

Additionally, Curthoys, Burgess, and Goonetilleke (2019) [18] demonstrated that irregular vestibular afferents exhibit exceptional temporal precision, with phase-locking capabilities extending beyond 1 kHz for bone-conducted and air-conducted stimuli. This ultrafast encoding capacity provides further physiological support for the specialization of Type I hair cells and their calyx-bearing afferents in the detection of rapid transients and jerk-like motion dynamics.

Importantly, the work of Cohen, Helwig, and Raphan [15] revealed that velocity storage is selectively engaged by low-frequency, sustained stimuli, while fast transient inputs bypass this integrator entirely—directly supporting the distinction between acceleration-sensitive (Type II/regular) and jerk-sensitive (Type I/irregular) pathways.

Similarly, Curthoys and Zee (2025) [6] demonstrated that clinical tests such as SVIN and vHIT preferentially activate the fast, irregular pathway while minimally engaging velocity storage, providing functional evidence that complements the mechanical and synaptic specializations highlighted in this review.

Together, these sources form an integrated literature scaffold that unites molecular zonation, multimodal synaptic transmission, dual-regime mechanics (viscous vs. inertial), and central integrative networks (including velocity storage and inhibitory circuitry) into a single, unified model of jerk encoding and dual-mode vestibular operation.

This review is particularly indebted to two very recent companion papers by Martin and colleagues, which provide the experimental foundation for much of the conceptual framework developed here. The first study, “The potassium channel subunit KV1.8 (Kcna10) is essential for the distinctive outwardly rectifying conductances of type I and II vestibular hair cells” (Martin HR, Lysakowski A, Eatock RA, eLife, 2024) [24], identifies KV1.8 as the key molecular determinant of gK,L in type I hair cells and of major components of gA and gDR in type II hair cells. The second study, “KV1.8 (Kcna10) potassium channels enhance fast, linear signaling in vestibular hair cells and facilitate vestibulomotor reflexes and balance” (Martin HR, Morris Verdone B, López-Ramírez O, Green M, Silvian D, Scott E, Cullen KE, Eatock RA, bioRxiv, 2025) [25], extends this work from ion-channel biophysics to synaptic transmission, vestibulo-ocular reflexes, and postural control. Together, these two articles offer a uniquely comprehensive link from molecular specialization (KV1.8) to temporal filtering properties of type I and type II pathways, and they form the primary empirical basis for the RC analogy and dual-mode (acceleration vs. jerk) framework discussed throughout this review. (see Appendix A for the RC-based interpretation of KV1.8 effects on τ and bandwidth).

## Figures and Tables

**Table 1:** Clinical vestibular tests and preferential activation of jerk- versus acceleration-dominated pathways.

Clinical Test	Stimulus Dynamics	Preferential Pathway	Velocity Storage	Key Clinical Use
Horizontal vHIT	High angular jerk	Canal Type I, irregular	Minimal	High-frequency canal deficit
Vertical canal vHIT	High vertical angular jerk	SSC/PSC Type I, irregular	Minimal	Vertical canal loss, plugging, dehiscence
Caloric irrigation	Very low-frequency convection	Canal Type II, regular	Strong	Hydrops, “caloric–vHIT dissociation”
Low-frequency rotational chair	Low-frequency angular velocity	Regular afferents + velocity storage	Strong	Bilateral hypofunction, central integration
Skull vibration-induced nystagmus	High-frequency skull vibration (jerk)	Mixed canals/otoliths, mainly Type I, irregular	Very limited	Jerk asymmetry, chronic unilateral loss
BCV oVEMP (≈500 Hz)	Transient BCV, linear jerk	Utricular striola Type I, irregular	Negligible	Superior vestibular nerve/utricle
BCV cVEMP (≈500 Hz)	Transient BCV, linear jerk	Saccule / inferior nerve Type I-rich	Negligible	Inferior vestibular nerve/saccule
ACS VEMPs	Acoustic bursts, mixed jerk + accel.	Otolith Type I + dimorphic, mixed discharge	Minimal	Frequency tuning, cochleo-vestibular links
Positional tests (Dix–Hallpike)	Near-static position change	Canal/otolith Type II, regular	Strong	Velocity storage, sustained tilt/rotation
Off-vertical axis rotation (OVAR)	Constant velocity + gravity modulation	Otolith + canal regular + velocity storage	Very Strong	Velocity storage, sustained tilt/rotation

## Data Availability

The original contributions presented in this study are included in the article. Further inquiries can be directed to the corresponding author.

## Abbreviations

The following abbreviations are used in this manuscript:

**ACS**	Air-Conducted Sound
**BCV**	Bone-Conducted Vibration
**CNS**	Central Nervous System
**Cyp26B1**	Cytochrome P450 Family 26 Subfamily B Member 1
**FSI**	Fluid–Structure Interaction
**GK,L**	Low-Voltage Activated Potassium Conductance in Type I Hair Cells
**HC**	Hair Cell
**HC I/HC II**	Type I Hair Cell/Type II Hair Cell
**HIT/vHIT**	(Video) Head Impulse Test
**HST**	Head Shaking Test
K*_LV_*	Low-Voltage Activated Potassium Channels in Afferents
**LDV**	Laser Doppler Vibrometry
**NaV1.6**	Voltage-Gated Sodium Channel Nav1.6
**NQT**	Nonquantal Transmission
**OKAN**	Optokinetic Afternystagmus
**OKN**	Optokinetic Nystagmus
**RA**	Retinoic Acid
**RC**	Resistor Capacitor
**SC**	Semicircular Canal
**SCC**	Semicircular Canal Complex
**SVIN**	Skull Vibration-Induced Nystagmus
**TC**	Time Constant
**UT**	Utricle
**VsEP**	Vestibular Short-Latency Evoked Potential
**VEMP**	Vestibular-Evoked Myogenic Potential
**VOR**	Vestibulo-Ocular Reflex

## Appendix A

RC Analogy: KV1.8, Time Constants and Bandwidth in Type I vs. Type II Hair Cells

In simple circuit terms, vestibular hair cells and their afferents can be viewed as RC elements whose membrane time constant

*τ* = *R*_in_⋅*C_m_*

sets both their temporal filtering in the time domain and their bandwidth in the frequency domain. The studies by Martin and colleagues on KV1.8 provide a direct experimental handle on this RC logic.

**Type I hair cells/irregular afferents: low-τ, high-bandwidth filter**

Type I hair cells express a very large low-voltage–activated K^+^ conductance (gK,L) that is critically dependent on KV1.8. This massive conductance lowers input resistance by more than an order of magnitude, yielding sub-millisecond membrane time constants (τ_RC ≈ 0.2–0.3 ms) and very high cutoff frequencies (f_c ≫ 100 Hz). In the RC analogy, Type I cells and their calyx afferents behave as low-impedance, fast-charging capacitors: they generate small-amplitude, rapidly changing receptor potentials that closely track the time course of jerk-dominated, inertial stimuli.

When KV1.8 is deleted (Kcna10^−^/^−^), gK,L disappears, input resistance increases by ~20-fold, and τ_RC expands into the millisecond range (≈4–6 ms). Consistent with the RC formula

*fc* ≈ 1/2*πτ*

the bandwidth of Type I responses collapses from the hundreds of hertz range down to ≈ 15–20 Hz, and receptor potentials become larger, slower, and more distorted. In other words, removing KV1.8 converts a fast, high-bandwidth differentiator into a much slower, more integrator-like element.

**Type II hair cells/regular afferents: high-τ, low-bandwidth filter**

Type II hair cells, by contrast, have smaller K^+^ conductances and higher input resistance. Their outward currents (g_A and g_DR) are partially KV1.8-dependent, but their overall conductance density is lower, yielding longer time constants and narrower bandwidth even in wild-type animals (cutoff frequencies on the order of tens of hertz). In RC terms, Type II cells behave as higher-τ filters that integrate sustained, viscous-flow–dominated acceleration.

In Kcna10^−^/^−^ mice, g_A is largely abolished, and a slower KV7-mediated g_DR becomes more prominent. The effective τ_RC increases further and the cutoff frequency drops (e.g., from ~70 Hz to ~20 Hz), reinforcing the slow, low-bandwidth character of the Type II pathway and enhancing its tendency toward resonance rather than rapid tracking of jerk.

**From RC parameters to functional specialization**

This RC analogy makes explicit how molecular specializations (KV1.8 vs. KV7) translate into different time constants (τ), which in turn determine bandwidth and phase. Type I/irregular pathways implement a low-τ, high-bandwidth temporal filter optimized for jerk-sensitive, inertial activation; Type II/regular pathways implement a high-τ, low-bandwidth filter optimized for sustained, acceleration-dominated motion. The “high- vs. low-frequency” behavior of these pathways is thus the frequency-domain expression of their underlying RC design.

## References

[1] Ono K., Keller J., López Ramírez O., González Garrido A., Zobeiri O.A., Chang H.H.V., Vijayakumar S., Ayiotis A., Duester G., Della Santina C.C. (2020). Retinoic acid degradation shapes zonal development of vestibular organs and sensitivity to transient linear accelerations. Nat. Commun..

[2] Contini D., Price S.D., Art J.J. (2017). Accumulation of K^+^ in the synaptic cleft modulates nonquantal transmission at the vestibular hair cell–calyx synapse. J. Physiol..

[3] Contini D., Holstein G.R., Art J.J. (2024). Simultaneous Recordings from Vestibular Type I Hair Cells and Their Calyceal Afferents in Mice. Front. Neurol..

[4] Grant W., Curthoys I. (2017). Otoliths-accelerometer and seismometer; implications in Vestibular Evoked Myogenic Potential (VEMP). Hear. Res..

[5] Goyens J., Pourquie M.J.B.M., Poelma C., Westerweel J. (2019). Asymmetric cupula displacement due to endolymph vortex in the human semicircular canal. Biomech. Model. Mechanobiol..

[6] Curthoys I.S., Zee D.S., Dumas G., Pastras C.J., Dlugaiczyk J. (2025). Skull vibration induced nystagmus, velocity storage and self-stability. Front. Neurol..

[7] Eatock R.A. (2025). Functional development and differentiation of mammalian vestibular hair cells and their synapses. Curr. Top. Dev. Biol..

[8] Pastras C.J., Curthoys I.S., Rabbitt R.D., Brown D.J. (2023). Using macular velocity measurements to relate parameters of bone conduction to vestibular compound action potential responses. Sci. Rep..

[9] Uchino Y., Kushiro K. (2011). Differences between otolith-and semicircular canal-activated neural circuitry in the vestibular system. Neurosci. Res..

[10] Uchino Y. (2004). Role of cross-striolar and commissural inhibition in the vestibulocollic reflex. Prog. Brain Res..

[11] Fernández C., Goldberg J.M. (1971). Physiology of peripheral neurons innervating semicircular canals of the squirrel monkey. I. Resting discharge and response to constant angular accelerations. J. Neurophysiol..

[12] Fernández C., Goldberg J.M. (1976). Physiology of peripheral neurons innervating semicircular canals of the squirrel monkey. II. Response to sinusoidal angular accelerations. J. Neurophysiol..

[13] Fernández C., Baird R.A., Goldberg J.M. (1988). The vestibular nerve of the chinchilla. I. Peripheral innervation patterns in the horizontal and superior semicircular canals. J. Neurophysiol..

[14] Goldberg J.M. (2000). Afferent diversity and the organization of central vestibular pathways. Exp. Brain Res..

[15] Cohen B., Helwig D., Raphan T. (1987). Baclofen and velocity storage: A model of the effects of the drug on the vestibulo—Ocular reflex in the rhesus monkey. J. Physiol..

[16] Cohen B., Dai M., Yakushin S.B., Cho C. (2019). The neural basis of motion sickness. J. Neurophysiol..

[17] Eatock R.A., Songer J.E. (2011). Vestibular hair cells and afferents: Two channels for head motion signals. Annu. Rev. Neurosci..

[18] Curthoys I.S., Burgess A.M., Goonetilleke S.C. (2019). Phase-locking of irregular guinea pig primary vestibular afferents to high-frequency (>250 Hz) sound and vibration. Hear. Res..

[19] Steinhausen W. (1927). Über die Beobachtung der Cupula in den Bogengangsampullen des Labyrinths des lebenden Fisches. Pflügers Arch. Ges. Physiol..

[20] Van Egmond A.A.J., Groen J.J., Jongkees L.B.W. (1949). The Mechanics of the Semicircular Canal. J. Physiol..

[21] Pastras C.J., Curthoys I.S., Asadnia M., McAlpine D., Rabbitt R.D., Brown D.J. (2023). Evidence that ultrafast nonquantal transmission underlies synchronized vestibular action potential generation. J. Neurosci..

[22] McGarvie L.A., Curthoys I.S., MacDougall H.G., Halmagyi G.M. (2015). What does the dissociation between the results of video head impulse versus caloric testing reveal about the vestibular dysfunction in Ménière’s disease?. Acta Oto-Laryngol..

[23] Curthoys I.S., Grant J.W., Burgess A.M., Pastras C.J., Brown D.J., Manzari L. (2018). Otolithic receptor mechanisms for vestibular-evoked myogenic potentials: A review. Front. Neurol..

[24] Martin H.R., Verdone B.M., Lopez-Ramirez O., Green M., Silvian D., Scott E., Eatock R.A. (2025). KV1.8 (Kcna10) potassium channels enhance fast, linear signaling in vestibular hair cells and facilitate vestibulomotor reflexes and balance. BioRxiv.

[25] Martin H.R., Lysakowski A., Eatock R.A. (2024). The potassium channel subunit KV1.8 (Kcna10) is essential for the distinctive outwardly rectifying conductances of type I and II vestibular hair cells. Elife.

[26] Curthoys I.S., Vulovic V. (2011). Vestibular Primary Afferent Responses to Sound and Vibration in the Guinea Pig. Exp. Brain Res..

[27] Curthoys I.S. (2017). The New Vestibular Stimuli: Sound and Vibration—Anatomical, Physiological and Clinical Evidence. Exp. Brain Res..

